# Modifiable Determinants of Postpartum Weight Loss in Women with Obesity: A Secondary Analysis of the UPBEAT Trial

**DOI:** 10.3390/nu13061979

**Published:** 2021-06-09

**Authors:** Kathryn V. Dalrymple, Onome Uwhubetine, Angela C. Flynn, Dharmintra Pasupathy, Annette L. Briley, Sophie A. Relph, Paul T. Seed, Majella O’Keeffe, Lucilla Poston

**Affiliations:** 1 Department of Women and Children’s Health, School of Life Course Sciences, King’s College London, London SE1 7EH, UK; onome.uwhubetine@kcl.ac.uk (O.U.); angela.flynn@kcl.ac.uk (A.C.F.); dharmintra.pasupathy@sydney.edu.au (D.P.); annette.briley@flinders.edu.au (A.L.B.); Sophie.relph@kcl.ac.uk (S.A.R.); paul.seed@kcl.ac.uk (P.T.S.); Lucilla.poston@kcl.ac.uk (L.P.); 2 Department of Nutritional Sciences, School of Life Course Sciences, King’s College London, London SE1 9NH, UK; majella.okeeffe@ucc.ie; 3 Westmead Reproduction and Perinatal Medicine Centre, Faculty of Medicine and Health, University of Sydney, Sydney 2006, Australia; 4 College of Nursing & Health Sciences, Flinders University, Adelaide 5042, Australia; 5 School of Food and Nutritional Sciences, University College Cork, T12 K8AF Cork, Ireland

**Keywords:** interventions, maternal obesity, postpartum weight retention, pregnancy, public health

## Abstract

Pregnancy can alter a woman’s weight gain trajectory across the life course and contribute to the development of obesity through retention of weight gained during pregnancy. This study aimed to identify modifiable determinants associated with postpartum weight retention (PPWR; calculated by the difference in pre-pregnancy and 6 month postpartum weight) in 667 women with obesity from the UPBEAT study. We examined the relationship between PPWR and reported glycaemic load, energy intake, and smoking status in pregnancy, excessive gestational weight gain (GWG), mode of delivery, self-reported postpartum physical activity (low, moderate, and high), and mode of infant feeding (breast, formula, and mixed). At the 6 month visit, 48% (*n* = 320) of women were at or above pre-pregnancy weight. Overall, PPWR was negative (−0.06 kg (−42.0, 40.4)). Breastfeeding for ≥4 months, moderate or high levels of physical activity, and GWG ≤9 kg were associated with negative PPWR. These three determinants were combined to provide a modifiable factor score (range 0–3); for each added variable, a further reduction in PPWR of 3.0 kg (95% confidence interval 3.76, 2.25) occurred compared to women with no modifiable factors. This study identified three additive determinants of PPWR loss. These provide modifiable targets during pregnancy and the postnatal period to enable women with obesity to return to their pre-pregnancy weight.

## 1. Introduction

The global prevalence of obesity has tripled over the last three decades [1]. This presents a major challenge for public health, as obesity is associated with comorbidities which impact quality of life and overall health, including hypertension and cardiovascular disease [2]. Excessive gestational weight gain (GWG) [3,4] with associated postpartum weight retention (PPWR) can contribute to the development of obesity in reproductive aged women [5,6]. Although many women return to their pre-pregnancy weight within 6 months of giving birth [7], longitudinal analyses suggest that women with obesity are at greater risk of major PPWR (>5 kg), being more likely to gain two or more BMI units and to move up to a higher BMI category compared to their healthy weight counterparts [8,9]. Therefore, pregnancy may alter weight gain trajectories across the life course and contribute to the development of obesity, through retention of gestational weight gained [10,11]. This also has adverse consequences for subsequent pregnancy outcomes [12].

In addition to pre-pregnancy BMI and excessive GWG, mode of delivery [13], ethnicity [14], socioeconomic deprivation [15], smoking [16], diet [17], physical activity levels [18,19] and short breastfeeding duration [20,21] have all previously been associated with PPWR in BMI heterogenous women. In the UK, the National Institute of Clinical Excellence recommendations [22] and evidence from two key systematic reviews [23,24] indicate that postnatal interventions including both diet and physical activity along with self-monitoring and frequent contact are more likely to be successful in promoting postpartum weight loss by 12 months. However, gaps were highlighted in relation to appropriate weight management interventions, particularly in women with obesity who are in greater need of effective weight management strategies. Identification of modifiable determinants that enable women to return to their pre-pregnancy weight will inform the development of targeted weight management interventions, which may influence maternal weight gain trajectories and reduce the prevalence of maternal obesity. These may include antenatal interventions for example to reduce GWG.

Despite the obvious benefit of recognising determinants of postpartum weight retention in women with obesity, the literature focusing on this group of women is very sparse. The aim of this study was, therefore, to identify antenatal and postnatal modifiable determinants associated with postpartum weight retention in women with obesity 6 months after giving birth, using data from the UK Pregnancies Better Eating and Activity Trial (UPBEAT).

## 2. Materials and Methods

UPBEAT was a randomised controlled trial (RCT) of an intensive 8 week antenatal diet and physical activity intervention compared to standard antenatal care. The study design and protocol were approved by the NHS Research Ethics Committee (UK Integrated Research Application System; reference 09/H0802/5). Full details of the trial were published previously [25]. In brief, 1555 women with a BMI ≥30 kg/m^2^, over the age of 16 years, with a singleton pregnancy were recruited to UPBEAT and randomised between 15^+0^ and 18^+6^ weeks gestation. The participants were recruited from eight UK inner-city centres from ethnically diverse and predominantly high socioeconomic-deprived backgrounds. The intervention aimed to prevent the development of gestational diabetes mellitus (GDM) and large for gestational age (LGA) infants through reducing dietary glycaemic load (GL) and saturated fat (SFA) intake, and by increasing physical activity levels compared to standard antenatal care. There was no effect of the intervention on the primary outcome of GDM and LGA infants. However, the intervention improved dietary intake, physical activity levels, total GWG, and maternal adiposity (all *p* < 0.04). Between July 2010 and June 2015, a planned follow-up appointment at 6 months postpartum of the women who participated in the UPBEAT was conducted. Women were excluded from this analysis if they were pregnant at the follow-up visit or gave birth at <37 weeks gestation.

### 2.1. Maternal Variables

The primary aim of this study was to identify determinants associated with women being at or below their pre-pregnancy weight at 6 months postpartum, assessed by PPWR, calculated as the difference in the mother’s pre-pregnancy weight, measured at baseline (15^+0^–18^+6^ weeks gestation) minus 1.25 kg, and the mother’s weight measured at the 6 month follow up appointment.

Maternal data were collected at three time points during pregnancy (baseline, 27^+0^–28^+6^ weeks and 34^+0^–36^+6^ weeks gestation) and at birth, at which point maternal and neonatal outcomes were collected. At baseline, demographic, medical, and family history information was collected. Socioeconomic deprivation was measured using the Lower Super Output Area (LSOA) and corresponds to the census region of the participant’s home postcode. This was subsequently converted to the corresponding index of multiple deprivation (IMD) quintiles and subscales. As the IMD scores are not directly comparable across England and Scotland, UK-wide scores were developed by reconciling Scottish data to English norms. At the three face-to-face antenatal visits, dietary intake and physical activity were assessed by a validated food frequency questionnaire (FFQ) and the International Physical Activity Questionnaire (IPAQ) [26], respectively. Body composition at these visits was assessed by weight, height, and skinfold thicknesses. Weight was measured without heavy clothing, shoes, and jewellery to the nearest 0.1 kg, and height was measured to the nearest cm without shoes using a portable stadiometer. After birth, mode of delivery and neonatal characteristics were recorded. At the 6 month follow-up visit, the FFQ and IPAQ questionnaires were repeated, maternal body composition was measured, and information on mode of infant feeding was recorded.

### 2.2. Exposure Variables

Exposures/modifiable determinants which have previously been associated with PPWR were included as exposure variables in the statistical analysis and included ethnicity, socioeconomic status, smoking status in pregnancy and postnatally (any vs. none), dietary intake (glycaemic load and total energy intake at baseline), antenatal and postnatal physical activity categorised as low (<600 metabolic equivalent task (METS)/week), moderate (600–2999 METs/week), and high (≥3000 METs/week), weight gain in accordance with the Institute of Medicine guidelines [27] categorised as inadequate (<5 kg), adequate (5–9 kg), and excessive (>9 kg), mode of delivery, and mode of feeding (exclusively breastfeeding ≥4 months or formula/mixed feeding). Four months was chosen as UK women commonly consider the introduction of complementary foods at this time [28]. Although dietary intake is a strong predictor for weight management, there were high rates of missing dietary data at 6 months postpartum (65.8% missing/incomplete questionnaires); therefore, dietary data were excluded as an exposure variable from the analysis.

### 2.3. Statistical Analysis

A complete case analysis was undertaken for all women who returned for the postpartum visit, where it was found there was no effect of the UPBEAT intervention on postpartum weight retention; therefore, the data were treated as a cohort. Summary statistics, as well as binary and categorical variables, are presented using counts and percentages. The distribution of continuous variables was assessed using coefficients of skewness and then summarised using mean and standard deviation with 95% confidence interval or median and interquartile range as appropriate. Using simple linear regression (model A), the association between the exposure variables and PPWR was quantified. Exposures with a significant association with PPWR (*p* < 0.05) were further examined using multiple linear regression (model B) with adjustment for confounders: maternal age, BMI at baseline, ethnicity, socioeconomic status, GDM, parity, gestational age at delivery, number of months since delivery, and randomisation arm. All analyses were performed in Stata version 15 (StataCorp, College Station, TX, USA).

#### Modifiable Factor Model for Postpartum Weight Retention

The secondary aim was to develop a modifiable risk model for the combined effects of the exposure variables associated with negative postpartum weight retention. The variables from model B which were significantly associated with negative PPWR (exclusive breastfeeding, GWG, and postpartum physical activity) were converted to binary variables, and a total score was generated (e.g., exclusive breastfeeding = 1, formula/mixed = 0; GWG ≤9 kg = 1, GWG >9 kg = 0, and moderate/high physical activity = 1, low physical activity = 0), with each woman being assigned an overall score. The score ranged from 0 to 3 (with 0 as the reference group). Using simple and multiple linear regression, the association with postpartum weight retention was examined on a continuous and categorical scale.

## 3. Results

**Figure 1:**
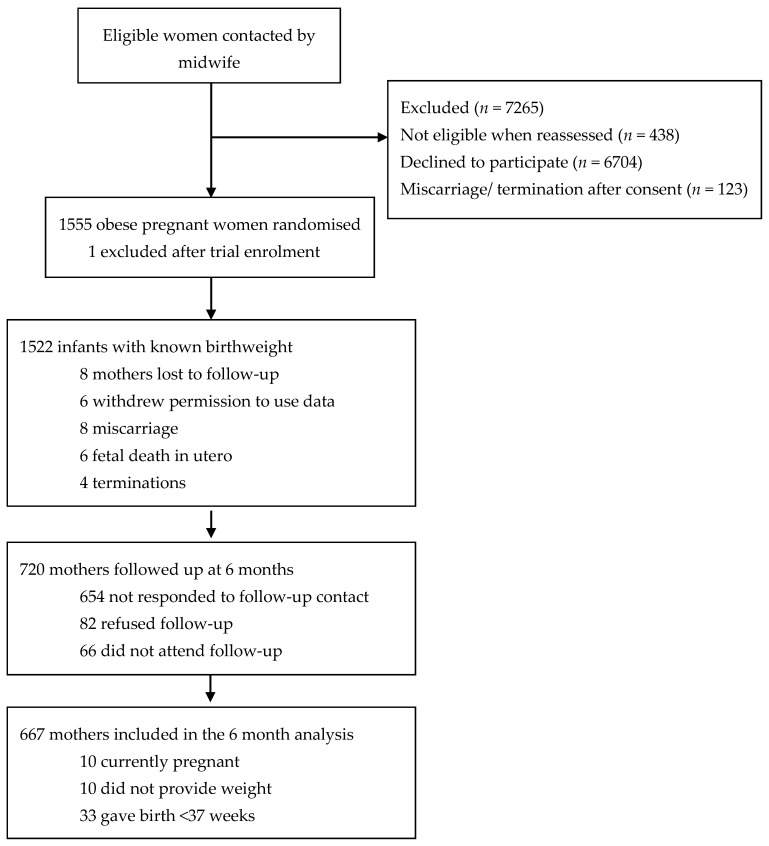
Consort diagram of participants enrolled in the UPBEAT trial at 6 months postpartum.

A total of 720 (46%) of the 1555 women from UPBEAT attended the follow-up visit at 6 months postpartum. At this visit, 10 women were pregnant, 11 had missing exposure or outcome data, and 32 gave birth at <37 weeks gestation; thus, they were excluded from this analysis. Therefore, the study population comprised 667 women (Figure 1).

### 3.1. Six Month Follow-Up

The average age at baseline was 31.2 years, the median BMI at baseline was 35 kg/m^2^ (IQR 32.7–38.7), 49% were nulliparous, 72% were White, and 77% had an Index of Multiple Deprivation category 4 or 5 (most deprived). The average GWG (15^+0^–18^+6^ weeks to 34^+0^–36^+6^ weeks gestation) was 7.14 kg, and, using the IOM guidelines for GWG, 30%, 34%, and 36% of women were categorised as having inadequate, adequate, and excessive GWG, respectively. Moreover, 27% of women had GDM in the index pregnancy (Table S1, Supplementary Materials), 40% of women had a caesarean delivery, and 64% were exclusively breastfeeding on hospital discharge (Table 1). In comparison to the UPBEAT women who did not take part, mothers who attended the 6 month visit were more likely to be Caucasian and nulliparous, were older at baseline, and had GDM in the index pregnancy [29].

Overall, the mean ± SD (range) PPWR was −0.06 kg ± 7.14 (−42.0 to 40.4 kg), and 52% (*n* = 347) of women were at or below their pre-pregnancy weight by 6 months postpartum. However, 21% (*n* = 145) of women retained ≥5 kg at 6 months postpartum. In the unadjusted model (model A), the IMD score indicating least deprivation, exclusive breastfeeding for ≥4 months, GWG ≤9 kg, and moderate or high levels of physical activity in the postpartum period were associated with negative postpartum weight retention (Table S2, Supplementary Materials). Being of Black or other ethnic origin, any smoking during pregnancy, and excessive GWG were each associated with weight retention (Table S2, Supplementary Materials). Dietary intake (glycaemic index and energy intake) at baseline was not associated with weight retention. After adjustment for confounders (model B), exclusive breastfeeding for ≥4 months, GWG ≤9 kg, and moderate or high levels of activity were associated with negative postpartum weight retention (Table 2).

### 3.2. Modifiable Factor Model for Postpartum Weight Retention

**Figure 2:**
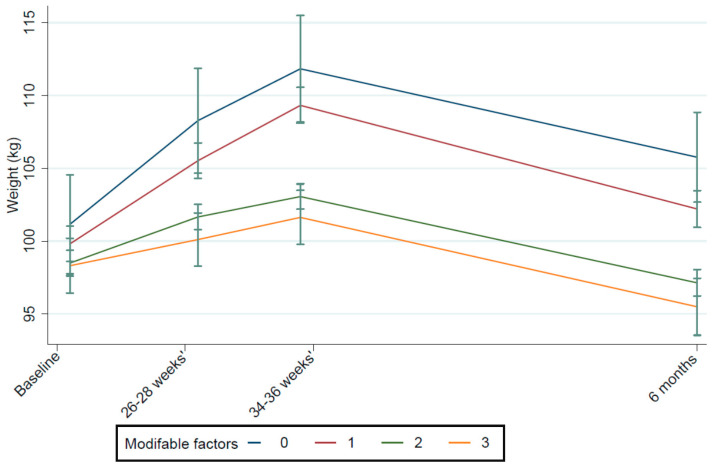
Mean weight from baseline to 6 months postpartum for women with zero, one, two, or three modifiable factors associated with negative PPWR. Notes: baseline = 15–18 weeks’ gestation, 6 months = 6 months postpartum. Factors include GWG ≤ 9 kg, breastfeeding ≥ 4 months, and moderate to high levels of physical activity in the postpartum.

Using the adjusted model, GWG ≤9 kg, exclusive breastfeeding for ≥4 months, and moderate or high levels of physical activity during the postpartum period were independently associated with negative postpartum weight retention. These three determinants were used in the model to assess any combined effect on postpartum weight. Women were categorised into the following groups: no modifiable determinants (3%, *n* = 19), one (30%, *n* = 183), two (51%, *n* = 314), and three (16%, *n* = 99) (Table 3). In these groups, the mean (SD) PPWR was +6.6 kg (6.84), +2.4 kg (7.18), −1.0 kg (6.18), and −3.2 kg (7.8), respectively. In an adjusted multivariate model, for each additional determinant, weight loss was −3.07 kg (95% confidence interval (CI): −3.79 to −2.35; *p* < 0.001) (Table 3, Figure 2). Compared to women who had no modifiable determinants, in women who displayed all three, there was a difference in weight of −9.52 kg (95% CI: −12.7 to −6.29; *p* = 0.01).

## 4. Discussion

The aim of this study was to identify antenatal and postnatal variables which contribute to postpartum weight loss in women with obesity. Description of these variables could inform interventions to prevent weight retention and exacerbate obesity in this high-risk group of women.

The findings demonstrate that women were more likely to return to their pre-pregnancy weight by 6 months after giving birth if they gained ≤ 9 kg of weight during pregnancy, undertook moderate to high levels of physical activity during the postpartum period, and exclusively breastfed for ≥4 months. We also observed positive, incremental associations between each determinant and postpartum weight loss, suggesting an additive influence of these modifiable variables.

Although the average PPWR retention was negative (−0.06 kg, range −42.0 to 40.4 kg), 21% of this cohort of women with obesity had major (>5 kg) postpartum weight retention. In contrast to previous reports, the average PPWR was lower than findings reported in similar sized cohorts of women with normal and overweight BMI [30,31]. These studies reported that ~70% of women retained some weight between 6 and 12 months after giving birth and over 30% retained > 5 kg. However, compared to the UPBEAT cohort, these trials included varying times of measurement of pre-pregnancy BMI including up to 2 years pre-conception; therefore, fluctuations in weight during the pre-conception period may have occurred. The finding that over half of the UPBEAT participants were already at their pre-pregnancy weight by the 6 month follow-up may reflect their inclusion in a randomised controlled trial which included accurate and continuous monitoring of weight during pregnancy by trained research staff.

The USA IOM guidelines report that excessive GWG is associated with PPWR [32]. This was also evident in the present study in which women with obesity who gained excessive GWG, i.e., over 30% of the cohort, retained +3.5 kg more than women with adequate gestational weight. Currently, in the UK, there is no guidance on weight gain during pregnancy other than avoidance of excessive GWG, and women are not routinely weighed during antenatal appointments. Our findings support the need for development of interventions to prevent excessive gestational weight gain in women with obesity in order to reduce postpartum weight retention.

Exclusive breastfeeding for ≥4 months, compared to formula or mixed feeding, was associated with mothers returning to their pre-conception weight. Recent findings from a prospective mother–infant cohort showed that breastfeeding for >3 months in weight heterogeneous women is associated with lower PPWR, compared to women who breastfeed for 1–3 months only. Furthermore, the authors highlighted that there was no difference in PPWR for mothers who breastfed from 3–6 months versus ≥6 months [33]. Viewed in conjunction with the present findings, interventions which promote breastfeeding past 3/4 months may increase the likelihood of women returning to pre-pregnancy weight. Our findings also add strength to the provision of targeted lactation support for women with obesity. Only 64% of the UPBEAT participants were exclusively breastfeeding on hospital discharge, significantly lower than the UK average of 81% [28]. Compared to normal weight women, women with obesity are less likely to initiate breastfeeding, and those who do often breastfeed for a shorter duration [34].

We also found an association between postpartum physical activity and weight loss. Until recently there were no UK guidelines for physical activity during the postpartum period [35]. A number of barriers impact postpartum physical activity including psychosocial factors, lack of time, energy, and availability of childcare [36]. A narrative review by McKinley et al. [20] summarised the current qualitative literature on barriers and exercise, including lack of partner support, motivation, confidence, and cost implications, which were higher in women from lower social classes. However, walking was highlighted as an affordable method which was perceived by women as a good form of postpartum exercise. This recommendation is included in the 2019 UK Chief Medical Officer’s physical activity guidelines [35]. Our findings report an association between moderate and high levels of activity and postpartum weight loss, which could encompass at least 30 min of walking for 5 or more days per week.

Several observational studies previously examined changes in weight across the postnatal period and identified factors predictive of PPWR [30,37,38]. However, the combined impact of these determinants has not previously been analysed in a cohort of obese women, in whom PPWR is a particularly important issue. The Southampton Women’s Survey reported that lower pre-pregnancy BMI, excessive GWG, lower early pregnancy vitamin D concentration, and breastfeeding for <6 months were associated with greater weight retention at 6 months postpartum in a group of women of heterogenous BMI [31]. However, the SWS cohort was >93% White with the majority of participants having a normal pre-pregnancy BMI. Although the exposures and the demographics of the cohort differ from the UPBEAT study, the two studies together provide a strong rationale for the relationship between PPWR loss and appropriate gestational weight gain and breastfeeding during the postnatal period. Furthermore, our results highlight a need to tailor weight management interventions according to BMI category, with women with obesity demonstrating additional barriers to weight loss than women with a healthier BMI. Additionally, socioeconomically deprived women have been largely underrepresented in previous studies, and women from areas of higher social deprivation often need more support, particularly with breastfeeding.

### Strengths and Limitations

A strength of this study lies in the longitudinal monitoring of maternal weight throughout pregnancy, measured accurately using calibrated scales and by a trained healthcare professional. The UPBEAT participants were from ethnically diverse and inner-city settings from across the UK. The study also included women of a low socioeconomic background with over 70% from the lowest quintiles of deprivation, providing data to inform development of interventions appropriate for these high-risk women. Lastly, adjustments were carried out for a broad range of confounding variables available in the UPBEAT dataset for inclusion in the statistics model. Limitations of the study include attrition of participants at the follow-up appointments. This is common in RCTs of mother–child dyads and may result in selection bias. However, as reported previously the maternal demographic characteristics at the follow-up appointment were similar to those of the initial trial cohort [29]. Smoking data were self-reported, and the physical activity data were obtained by the self-reported IPAQ, which is subject to bias. The IOM guidelines were used to define GWG, which were devised using data from the United States population and may not be transferable to UK women.

## 5. Conclusions

The antenatal and postnatal periods are times of heightened health awareness when women may be more receptive to lifestyle modifications [39]; they are, therefore, an opportune time to implement interventions which focus on developing lifelong healthy behaviours. The postpartum period is also classed as the preconception period for a subsequent pregnancy, and positive health changes are likely to benefit future pregnancy health. Based on this study, we found an incremental reduction in weight retention for each additional factor associated with postpartum weight loss. Initiatives which target gestational weight gain, breastfeeding duration, and postpartum physical activity could help prevent women with obesity retaining weight postnatally. With rising trends in the development of obesity, effective and evidence-based strategies are needed to support women to return to their pre-pregnancy weight. Here, we identified targets suitable for public health interventions for women with obesity.

## Figures and Tables

**Table 1:** Maternal and infant demographics included in the analysis.

Maternal Demographics		*N*	Mean (SD)/Median (IQR)/*N* (%)
**15–18 weeks gestation (baseline)**			
Age at baseline (years)		667	31.2 (5.3)
BMI (kg/m^2^)		667	35 (32.7–38.7)
Nulliparous		667	330 (49)
Ethnicity	Asian	667	23 (3)
	Black		125 (19)
	Other		42 (6)
	White		477 (72)
Any cigarette smoking during pregnancy		667	92 (14)
SES (IMD score) ^¥^	1 (least deprived)	665	31 (5)
	2		47 (7)
	3		74 (11)
	4		242 (36)
	5 (most deprived)		271 (41)
Years in full time education		667	15.1 (2.84)
**Infant demographics**			
Gestation at delivery (days)		667	279 (9.0)
Mode of delivery	LSCS in labour	667	128 (19)
	Operative vaginal		90 (14)
	Prelabour LSCS		139 (21)
	Unassisted vaginal		310 (46)
Neonate birthweight (g)		667	3525 (474)
Macrosomia (>4 kg)		667	101 (15)
LBW (<2.5 kg)		667	9 (1)
Mode of feeding at hospital discharge	Breastfeeding	665	425 (64)
	Formula feeding		133 (20)
	Mixed feeding		107 (16)
**Maternal antenatal and postpartum characteristics**			
Gestational diabetes mellitus **		656	181 (27)
Gestational weight gain (kg) ^¶^		641	7.39 (4.52)
GWG according to IOM Guidelines ^¶^	Inadequate	639	194 (30)
	Adequate		217 (34)
	Excessive		230 (36)
Exclusively breastfeeding ≥ 4 months		621	187 (30)
Postpartum weight retention (kg)		667	−0.06 (7.14)
PPWR >5 kg		667	145 (21%)
Physical activity reported at 6 months (METs/week) ^†^	Low	666	92 (14)
	Moderate		329 (49)
	High		245 (37)

Abbreviations: BMI: body mass index; IOM: Institute of Medicine Guidelines; LBW: low birth weight; LSCS: lower (uterine) segment caesarean section; METs: metabolic equivalent task; SES: socioeconomic status; ^¥^ IMD quintiles calculated for the region of residence, by fifths of the population. UK-wide scores were developed by reconciling Scottish data to English norms. ** Gestational diabetes diagnosis by International Association of Diabetes in Pregnancy Study Group criteria at 27^+0^ to 28^+6^ weeks gestation. ^¶^ Gestational weight gain calculated using estimated weight before pregnancy according to the IOM Weight Management in Pregnancy Guidelines’ category. ^†^ MET is defined as the energy expenditure ratio of activity to rest; 1 MET is approximately equal to an individual’s resting energy expenditure.

**Table 2:** Adjusted multiple linear associations between significant protective factors from model A and postpartum weight retention (continuous outcome).

Factors	*N*		B-Coefficient 95% CI *	*p*-Value
Smoking in pregnancy	648		1.33 (−0.17 to 2.84)	0.083
Breastfeeding ≥4 months	603		−2.33 (−3.55 to −1.11)	<0.0001
IOM GWG ^¶^	193	Inadequate	−2.06 (−3.32 to −0.80)	0.001
	216	Adequate	Ref	
	229	Excessive	3.47 (2.26 to 4.67)	<0.0001
Postpartum physical activity (METs/week) ^†^	109	Low	Ref	
	276	Moderate	−1.62 (−3.19 to −0.05)	0.043
	278	vigorous	−1.75 (−3.41 to −0.09)	0.039

Abbreviations: GWG: gestational weight gain; IOM: Institute of Medicine Guidelines; METs: metabolic equivalent task. * Adjusted for parity, maternal BMI at baseline, ethnicity, IMD score, GDM diagnosis, maternal age, gestational age at delivery, months since birth, and randomisation arm. ≥ greater than or equal to, ^¶^ Gestational weight gain calculated using estimated weight before pregnancy according to IOM Weight Management in Pregnancy Guidelines’ categories. ^†^ MET is defined as the energy expenditure ratio of activity to rest; 1 MET is approximately equal to an individual’s resting energy expenditure.

**Table 3:** Postpartum weight retention at 6 months in women according to the number of protective factors.

		Unadjusted (*n* = 595)		Adjusted (*n* = 587)	
Factors (*N* (%))	Mean (SD) PPWR	B-Coef. 95% CI	*p*-Value	B-Coef. 95% CI	*p*-Value
0 (19 (3%))	+6.6 kg (6.85)	Ref		Ref	
1 (183 (30%))	+2.4 kg (7.18)	−4.21 (−7.52 to −0.92)	0.012	−3.72 (−7.06 to −0.38)	0.029
2 (314 (51%))	−1.0 kg (6.18)	−7.65 (−10.8 to −4.42)	<0.000	−7.02 (−10.3 to −3.73)	<0.000
3 (99 (16%))	−3.1 kg (7.81)	−9.81 (−13.2 to −6.39)	<0.000	−9.38 (−12.7 to −5.89)	<0.000
Β- trend		−3.06 (−3.80 to −2.31)	<0.000	−3.00 (−3.76 to −2.25)	<0.000

Adjusted for, smoking status, maternal age and BMI at baseline, ethnicity, socioeconomic status, parity, GDM status in pregnancy, randomisation arm, gestational age at delivery, and months since giving birth. Women were excluded if they gave birth before 37 weeks gestation and were pregnant at the follow-up visit. Protective factors included GWG ≤ 9 kg, breastfeeding for > 4 months, and moderate/high levels of postnatal physical activity. Ref = reference category.

## Data Availability

The data presented in this study are available on request from the corresponding author.

## Supplementary Materials

The following are available online at https://www.mdpi.com/article/10.3390/nu13061979/s1: Table S1. Additional maternal and infant demographics; Table S2. Unadjusted associations between factors associated with postpartum weight retention (A).
