# Supplementary material for: Modifiable Determinants of Postpartum Weight Loss in Women with Obesity: A Secondary Analysis of the UPBEAT Trial
Source: Nutrients. 2021 Jun 9;13(6):1979. doi: 10.3390/nu13061979 (PMC8227672; doi:10.3390/nu13061979)
Supplement: Supplementary file 1 [file nutrients-13-01979-s001.zip › nutrients-1224763-supplementary.pdf]

Supplementary Table S1: Additional Maternal and infant demographics

**Maternal demographics**		**N**	**Mean (SD)/ Median (IQR)/ N%**
**15-18 weeks' gestation (baseline)**			
WHO BMI categorises			
Obese I (30.0-34.9 kg/m^2)			331 (50)
Obese II (35.0 -39.9 kg/m^2)			207 (31)
Obese III (≥40.0 kg/m^2)			129 (19)
Intervention		667	330 (49)
Glycaemic index		578	56.5 (4.2)
Energy intake (Kcal)		578	1716 (766)
Physical activity (METs/week) †	Low	578	139 (24)
	Moderate		300 (52)
	High		139 (24)
**Infant demographics**			
Large for gestational age >90th centile for gestational age*		667	86 (13)
**Maternal antenatal and postpartum characteristics**			
Gestational diabetes mellitus**		656	181 (27)
Exclusively breastfeeding ≥ four months		621	187 (30)

*Abbreviations: BMI: body mass index; METs: Metabolic equivalent task; SES: Socio-economic Status; WHO: World Health Organisation * Customised birthweight centile calculated adjusting for maternal height and weight, ethnic origin, parity and sex of the infant. †MET is defined as the energy expenditure ratio of activity to rest; 1 MET is approximately equal to an individual's resting energy expenditure*

Supplementary Table S2: unadjusted associations between predictors associated with postpartum weight retention (model A)

Predictor		*B-coefficient* 95% CI	*p-value*
**Maternal characteristics**			
Ethnicity	*White*	Ref	0.056
	Black	1.36 (0.03 to 2.70)	
	Asian	0.11 (-2.72 to 2.94)	
	Other	2.32 (0.16 to 4.48)	
Smoking at all in pregnancy		1.97 (0.48 to 3.45)	0.01
IMD[¥]	*1 (Least deprived)*	-2.65 (-5.21 to -0.10)	0.28
	2	-0.82 (-2.92 to 1.28)	
	3	-0.88 (-2.63 to 0.86)	
	4	-0.17 (-1.35 to 1.00)	
	5 (Most deprived)	Ref	
**15-18 weeks' gestation**			
Glycaemic Index		-0.07 (-0.21 to 0.06)	0.26
Energy intake (kcal)		-0.0002(-0.0009 to 0.0005)	0.63
Intervention		-0.76 (-1.84 to 0.32)	0.17
**Infant demographics**			
Mode of delivery	*Unassisted vaginal*	Ref	0.07
	LSCS in labour	0.28 (-1.11 to 1.67)	
	Operative vaginal	0.84 (-0.75 to 2.43)	
	Pre-labour LSCS	-1.35 (-2.71 to 0.0004)	
Breast feeding ≥ 4months		-2.14 (-3.33 to -0.96)	<0.001
**Maternal antenatal and postnatal characteristics**			
NAM GWG[¶]	*Inadequate*	-2.23 (-3.49 to -0.98)	<0.001
	Adequate	Ref	
	Excessive	3.53 (2.33 to 4.72)	
Smoking postpartum		1.74 (-0.28 to 3.77)	0.09
Postpartum physical activity (METs/week)	*Low*	Ref	0.04
	Moderate	-1.90 (-3.51 to -0.28)	
	Vigorous	-1.91 (-3.58 to -0.24)	

Abbreviations: NAM: National Academy of Medicine Guidelines; GWG: gestational weight gain; METs: Metabolic equivalent task; SES: Socio-economic Status; [¥]IMD quintiles are calculated for the region of residence, by fifths of the population. UK wide-scores were developed by reconciling Scottish data to English norms. [¶]Gestational weight gain calculated using estimated weight before pregnancy according to the NAM Weight Management in Pregnancy Guidelines. [†]MET is defined as the energy expenditure ratio of activity to rest; 1 MET is approximately equal to an individual's resting energy expenditure
